# The Processes of Change Scale: A Confirmatory Study of the Malay Language Version

**DOI:** 10.21315/mjms2020.27.3.13

**Published:** 2020-06-30

**Authors:** Aizuddin Hidrus, Yee Cheng Kueh, Norsa’adah Bachok, Garry Kuan

**Affiliations:** 1 Biostatistics and Research Methodology Unit, School of Medical Sciences, Universiti Sains Malaysia, Malaysia; 2 Community and Family Medicine Department, Faculty of Medicine and Health Science, Universiti Malaysia Sabah, Malaysia; 3 Exercise and Sports Science, School of Health Sciences, Universiti Sains Malaysia, Kelantan, Malaysia; 4 Department of Life Sciences, Brunel University London, United Kingdom

**Keywords:** validity, reliability, confirmatory factor analysis, university students, psychometric evaluation

## Abstract

**Background:**

Processes of change (POC) comprise one of the psychological constructs in the Transtheoretical Model. The objective of this study is to test the validity and reliability of the Malay version of the POC scale among university students by using a confirmatory approach.

**Method:**

A cross-sectional study design with a convenience sampling method using a self-administered questionnaire was carried out. University undergraduate students were approached to fill in the questionnaire, which consisted of demographic information and a POC scale. The POC scale consisted of 30 items and two main factors (i.e., cognitive and behavioural). The POC scale was translated into the Malay language using a standard procedure of forward and backward translation. Confirmatory factor analysis (CFA) was performed, and composite reliability was computed using Mplus version 8.

**Results:**

A total of 620 respondents with a mean age of 20 years (standard deviation = 1.15) completed the questionnaire. Most of the participants were female (74.7%) and Malay (78.2%). The initial CFA model of the POC scale did not exhibit fit based on several fit indices (comparative fit index (CFI) = 0.880, Tucker Lewis index (TLI) = 0.867, standardised root mean square residual (SRMR) = 0.075 and root mean square error of approximation (RMSEA) = 0.058). Several re-specifications of the model were conducted and the modification included adding correlation between the items’ residuals. The final model for the Malay version of the POC scale showed acceptable values of model fit indices (CFI = 0.922, TLI = 0.911, SRMR = 0.064 and RMSEA = 0.048). The composite reliability of both the cognitive and behavioural processes was acceptable at 0.856 and 0.752, respectively.

**Conclusion:**

The final model presented acceptable values of the goodness of fit indices, indicating that the scale is fit and acceptable to be adopted for future study.

## Introduction

The transtheoretical model (TTM), also known as the stage of change (SOC) model, finally matured in the 1990s (1). According to Kim (2), “TTM accounts for the dynamic nature of health behaviour change including exercise, and recognises that individuals often must make several attempts at behaviour change before they are successful.” TTM consists of four core constructs: A. The six stages of exercise behaviour change (3) include i) pre-comtemplation; ii) contemplation; iii) preparation; iv) action; v) maintenance and vi) relapse; B. The psychological constructs, include processes of change (POC) (consisting of overt and covert activities that individuals utilise to modify their behaviour); C. Decisional balance (involving the perceived ‘pros’ (advantages) and ‘cons’ (disadvantages) of continuing a current behaviour or adopting a new behaviour) (4); and D. Self-efficacy (how well one can execute the courses of action required to deal with prospective situations) (5).

Processes of change comprise one of the psychological constructs in TTM, which was developed by Prochaska and DiClemente (6). It is one of the most well-known motivational models used to promote physical activity (PA) programmes (7). Kim (2) has defined TTM as “a contemporary psychological framework that attempts to explain intentional health behaviour adoption and maintenance as a process that occurs over time as a function of behavioural history and motivation.” POC has been used along with other TTM constructs for examining the factors influencing the amount of PA performed among university and school students (8, 9).

Society has adopted POC as a scheme or method to adjust their self-developed behaviour (3). POC can be classified into either experiential or cognitive processes or behavioural processes (2, 10). Experiential processes are comprised of consciousness raising, dramatic relief, self-reevaluation, environmental reevaluation and self-liberation that are derivative of an individual’s own actions, while behavioural processes’ information is obtained through environmental events, including social liberation, counter conditioning, stimulus control, reinforcement management and helping relationships.

For decades, PA and exercise have been empirically accepted by clinicians and researchers as improving the health status of the general population. The World Health Organization (WHO) stated that physical inactivity is one of the major causes of various types of non-communicable diseases (11). Since then, countries around the world have been promoting exercise to reduce the prevalence of morbidity and mortality due to physical inactivity. Despite this, not only is the adult or elderly population limited with respect to PA levels, but a high number of studies have reported results showing that most of the world’s youth population is also physically inactive (12, 13).

In Malaysia, reports from National Health and Morbidity 2017 showed that only 45% of Malaysian students are physically active (14). Thus, aside than the existing methods, new approaches and instruments should be brought in to improve the prevalence of PA among students in Malaysia. With this in mind, studies have shown that POC could predict an individual’s PA behaviour (15–17). Previous studies have also emphasised that both experiential and behavioural processes are required for someone who is trying to begin, improve or sustain in PA (18–21). Therefore, by applying POC in the context of assessing our Malaysian students, we could potentially predict their PA behaviour. Researchers and healthcare policy makers could therefore improve Malaysian students’ PA participation with more suitable and effective methods of intervention that target individuals’ cognitive and behavioural processes.

Although one study has tested the validity of the short version of the POC scale among Malaysian primary school children (9), the full POC scale in the Malay language has not been tested among Malaysian adults. Considering that the POC is relatively new in Malaysia, we decided to translate the English version of the POC scale into the Malay language. Hence, the scale can still be used for university or college students, but it can also be used to assess the POC of primary and secondary students as well. Therefore, the present study aimed to test the validity and reliability of the Malay version of the POC scale among university undergraduate students in the Universiti Sains Malaysia using a confirmatory approach.

## Materials and Methods

### Study Design, Procedures and Participants

Data collection was held in Health Campus of Universiti Sains Malaysia, Kubang Kerian, Kelantan. A cross-sectional study design with convenience non-probability sampling method was carried out among the university undergraduate students from March to April 2019. Convenience sampling was used due to its simplicity in recruiting participants and data collection takes minimal time. Large sample size is preferable for confirmatory factor analysis (CFA), thus this sampling method allowed researchers to obtain enough number of participants in completing the questionnaire. University students who volunteered were undergraduate students from different courses, which include medicine, nursing, dental, sports science, biochemistry and others. The students were approached to participate in this study after their lecture at the university. The participants were briefed on the current study and those who wish to participate were required to answer the self-administered questionnaire. The participants took between 15 min and 20 min to complete the questionnaire.

### Instrument

The questionnaire consists of two sections, the demographic and the POC scale sections. The demographic section, information such as age (years), gender, ethnicity, BMI, PA duration per week were collected.

### POC

The scale was developed by Nigg et al. (22). The 30-items questionnaire is rated on a 5-point Likert scale ranging from 1 (never) to 5 (repeatedly). It measures the 10 factors: consciousness raising, dramatic relief, selfreevaluation, environmental reevaluation, social liberation, counter-conditioning, helping relationships, reinforcement management, stimulus control and self-liberation. The internal consistency reliability was reported to be 0.6 and 0.9 for the two higher factors, cognitive and behavioural processes, respectively (22).

### Translation of POC Scale into Malay Language

The original English version of the POC scale was translated into the Malay language using the forward and backward standardised procedures outlined by Brislin (23). The steps include: i) one of the bilingual authors familiar with the content did the forward translation into Malay language, based on the principle of retaining meaning, rather than literal word-for-word translations; ii) another bilingual expert back-translated the Malay version into English language; iii) three panels consisted of experts from the areas of sport psychology, sport sciences, psychometric evaluation with over 10 years of experience in their areas of expertise, reviewed and examined the forward and backward translated versions.

All panels were competent bilingual languages, both in Malay and English. They reviewed the English translation from the Malay version and the Malay translation from English, comparing each item to the corresponding item on the original English version. They noted any deviations in meaning and finalised the Malay version of POC scale (POC-M). Then, expert panels were asked to assess whether the contents were culturally appropriate to the Malaysian population. The final version of POC-M was pre-tested among 30 university students for clarity, comprehension and understanding. The result of the pre-test among the university students was found to be acceptable, and no modifications were necessary. The POC-M was used in the present study.

### Data Analysis

CFA was performed by using Mplus version 8. Demographic data of participants were presented by descriptive information. For categorical variables, they were presented by frequencies and percentage, whereas for numerical variables, they were presented by mean and standard deviation (SD).

The data were checked for multivariate normality, and the results indicated that the data did not meet the assumption, based on Mardia multivariate skew (*P* < 0.001) and kurtosis (*P* < 0.001) tests. Therefore, for the subsequent CFA, the robust maximum likelihood estimator (MLR) was utilised, as this is robust to non-normality (24–26). Researchers had suggested presenting more than one fit index in order to show the validity of the questionnaire (27). Based on 30-item of POC-M measurement model in the present study, the fit indices and its acceptable threshold value are as follows: the comparative fit index (CFI) and Tucker and Lewis index (TLI) with the desired value of more than 0.92; the root mean square error of approximation (RMSEA) with the desired value of less than 0.08; probability RMSEA with the desired value of more than 0.07; and the standardised root mean square (SRMR) with the desired value of less than 0.08 (28).

Modification of the model may be required to achieve the recommended cut-off values for each model fit indices. Modification includes removing the items with low factor loading and adding correlation between items’ residual. An item with factor loading less than 0.4 was treated as problematic items (29, 30) and subjected for removal after adequate theoretical support was carried out. Correlation between items’ residual in the same factor could be added in the model to improve the fit indices. To access the convergent validity of the scale, average variance extracted (AVE) and composite reliability (CR) of the final measurement model were computed.

### Sample Size

In CFA, larger samples generally produce more stable solutions and are more likely to be replicable. Based on Hair et al. (28), with a number of factors larger than six, sample size requirements may exceed 500. In the present study, POC-M consists of more than six factors, so we considered that the sample size of 620 was sufficiently large for a confirmatory study using CFA.

## Results

### Demographic Characteristics

A total of 620 volunteered and participated in the present study. Male students were 157 (25.3%), while female students were 463 (74.7%), with a mean age of 20 (1.15). Among the 620 students, 485 (78.2%) were Malay, 97 (15.6%) were Chinese, 29 (4.7) were Indian and 9 (1.5%) were others. Participants had mean duration of exercise per week of 53 min (SD = 33.4).

### Descriptive Statistics

Table 1 shows the distribution of the items’ score answered by the students. As a whole, the majority of students answered ‘occasionally’ for all items. For ‘never’ score, the lowest number of student answered is item PC11 (*Saya percaya bahawa senaman yang tetap akan membuat saya menjadi lebih sihat dan lebih gembira.*), indicating most of the students are believing that PA could lead them to a healthier and happier lifestyle. Item PC29 (*Saya menggunakan kalendar untuk menetapkan waktu senaman saya.*) was the highest answer on ‘never’ score, as probably most of the students prefer to do PA during their leisure time. Highest and lowest answered for ‘repeatedly’ was item PC11 and PC29 respectively, prove that students are assured with the PA advantages towards health status, but practice it only during their free time. Noticed that there was a ceiling effect on the item PC29 which most of the students answered ‘never’. However, the rest of the items’ score were normally distributed and no ceiling and floor effect found in the Malay version of the POC scale.

## Confirmatory Factor Analysis

The POC-M consists of 30 items with ten first-order factors (consciousness-raising, dramatic relief, self-reevaluation, environmental reevaluation, self-liberation, social liberation, counter-conditioning, stimulus control, reinforcement management, and helping relationships) that divided into the second-order factors (experiential/cognitive and behavioural processes). In the initial hypothesised measurement model (initial), the factor loading of all items was higher than 0.4 (Table 2).

However, two of the model fit indices were not in the acceptable value (CFI = 0.880, TLI = 0.867, SRMR = 0.075, RMSEA = 0.058) (Table 3). As the items’ factor loading were all higher than 0.4, there was no item need to be removed. Thus, further investigation of the initial model output was carried out and found that, there were numbers of correlation between items’ and first factors’ residuals with high value (higher than 10) in the output of the initial model.

As mentioned before, the addition of these correlation residuals in the model could improve the model fit indices; thus, we decided to include the correlation residuals into the model. Addition of the correlation residuals has been done iteratively, started with the highest value to the lower value until the model fit the data. Final model with the accepted value of goodness of fit indices was achieved after 11 correlation between items’, and first-order factors’ residuals were added (Table 3).

Composite reliability (CR) of the second-order factors in the final model was calculated based on Raykov’s method (31). There are two opinions for the cut-off point of composite reliability, > 0.6 by Tseng et al. (32) and > 0.7 by Hair et al. (28) The second-order factors’ composite reliability for the final model were 0.85 for cognitive processes and 0.86 for behavioural processes (Table 4). The AVE values were also provided in Table 4. Both factors had AVE values more then 0.50.

## Discussion

Through the adoption of CFA, the current study was aimed to present the validity and reliability of POC-M scale among undergraduate students in the Health Campus of Universiti Sains Malaysia. After the addition of 11 correlations between items and second-order factors residuals into the model, we finally obtained the best model fit indices. There were no problematic items with lower than 0.4 items’ loading found. Hence, no item was removed to achieve the required model fit indices value.

From the result, we can see that there were more female participants (74.7%) than male participants (25.3%). This can be explained by the typical situation of the students’ population in local universities where females are commonly higher in number compared to males. Moreover, as the questionnaire were requested among the students who voluntarily to participate, female students were more co-operative compared to males. Regarding the obtained model fit indices value for the present study, showed that all model fit indices were met the suggested value by Hair et al. (28) except for TLI. Nonetheless, the acceptable values for model fit indices are vary based on the viewpoint of different researcher all around the globe. As an example, the cut-off point for CFI (33) was initially 0.9. After did some revision, Hu and Bentler (34) were then increased the cut-off point for CFI ad TLI by 0.95 or higher. Another opinion given by McDonald and Ho (35) stated that the value of higher than 0.9 for CFI is acceptable. Guideline of model fit indices designed by Hooper et al. (36) exhibit that 0.8 is the value as a recommended cut-off point for TLI, also known as NFI (normed-fit index). Hence, the obtained value of model fit indices for the present study is considered as in the acceptable value and indicate the model fits the data well.

Initially, POC was commonly adopted in assessing smoking cessation among adults (37, 38). Back then, it was difficult to find a study that applies the TTM, specifically POC on adults/ adolescent population related to PA other than a study by Nigg and Courneya (39). Until now, we are able to find a few numbers of studies adopting POC scale on PA behaviour towards college students (40), adolescents (41) and African American (42). The development of POC scale for exercise behaviour was initially with 40 items and validated in the English language. It was then revised and simplified into 30 items (22). As it widely adopted all around the world, POC scale for PA has been translated into different languages such as, Iranian (43), Korean (2), Finnish (44) and French (45).

As for the French language (45), two different studies were carried out. In the first study, item/scale descriptive statistics performed and all 30 items were reserved. For the second study, five models were measured with each of the models consist of different structure: Model A (ten first-order factors subordinate to two correlated second-order factors), Model B (the ten fully correlated factors model), Model C (the two second-order factors and eight first-order factors), Model D (the nine first-order factors subordinate to two correlated second-order factors), and Model E (the five fully correlated factors measurement model), among 671 participants through online and paper-and-pencil approach. As the main objective is to compare the existing models in order to validate the French POC scale, multiple CFA were implemented. Final results showed that only Model A and Model E presented moderate fit indices with the data. Model E was the best model with Χ^2^ = 653.46, CFI = 0.920, SRMR = 0.056 and RMSEA = 0.060.

Similar to other studies, the present study also faced some limitations. As the participants were only undergraduate students of Health Campus of Universiti Sains Malaysia; thus, the generalisability of the present study are limited. Although all Malaysians could read and understand the Malay language well, yet, different layers of age, socio-economic and educational backgrounds, and other factors may lead to different understanding and interpretation of the questionnaire items. Insincerity and dishonesty could bring biases when self-administered approach applied. This may lead to the violation of the instrument reliability; however, before students were encouraged to answer the questionnaire sincerely and honestly. They were also asked to answer the questionnaire without discussing with other participants. All of these precautions were applied as the best way to reduce bias. Based on gender and ethnicity, participants were dominated by female and Malay. Therefore, the invariance test between genders and ethnicities could not be carried out. Future research should be conducted with better sampling method which could get balance participants between male and female; and different ethnicities.

## Conclusion

The objective of the current study on validating the Malay version of the POC scale was achieved. The final model presented the acceptable value of goodness fit of indices, indicating that the scale is fit and acceptable to be adopted among Malaysian university students. Thus, it can be beneficial in assessing POC towards the PA of target population. Nonetheless, it is highly suggested for future study to identify better sampling method in order to collect a broader representative of the population. More universities, colleges, and schools should be involved if the target population are students. Variety of socio-economic and educational backgrounds, rural and urban area and different layers’ age also need to be concerned in order to produce better study sample. Hence, results from the future study will be more comprehensive and could be generalised to all Malaysian populations.

## Figures and Tables

**Table 1:** Distribution of the items’ score for POC-M

Items	Score				

	Never *n* (%)	Seldom *n* (%)	Occasionally *n* (%)	Often *n* (%)	Repeatedly *n* (%)
PC1	38 (6.1)	181 (29.2)	272 (43.9)	106 (17.1)	23 (3.7)
PC2	38 (6.1)	165 (26.6)	273 (44.0)	111 (17.9)	33 (5.3)
PC3	51 (8.2)	155 (25.0)	245 (39.5)	140 (22.6)	29 (4.7)
PC4	65 (10.5)	150 (24.2)	235 (37.9)	130 (21.0)	40 (6.5)
PC5	22 (3.5)	86 (13.9)	209 (33.7)	202 (32.6)	101 (16.3)
PC6	110 (17.7)	104 (16.8)	208 (33.5)	148 (23.9)	50 (8.1)
PC7	46 (7.4)	94 (15.2)	230 (37.1)	169 (27.3)	81 (13.1)
PC8	36 (5.8)	88 (14.2)	225 (36.3)	213 (34.4)	58 (9.4)
PC9	21 (3.4)	71 (11.5)	222 (35.8)	219 (35.3)	87 (14.0)
PC10	12 (1.9)	70 (11.3)	201 (32.4)	239 (38.5)	98 (15.8)
PC11	4 (0.6)	52 (8.4)	192 (31.0)	237 (38.2)	135 (21.8)
PC12	12 (1.9)	44 (7.1)	203 (32.7)	242 (39.0)	119 (19.2)
PC13	18 (2.9)	70 (11.3)	188 (30.3)	238 (38.4)	106 (17.1)
PC14	15 (2.4)	77 (12.4)	203 (32.7)	243 (39.2)	82 (13.2)
PC15	19 (3.1)	90 (14.5)	204 (32.9)	233 (37.6)	74 (11.9)
PC16	44 (7.1)	181 (29.2)	234 (37.7)	128 (20.6)	33 (5.3)
PC17	79 (12.7)	200 (32.3)	201 (32.4)	106 (17.1)	33 (5.3)
PC18	53 (8.5)	154 (24.8)	257 (41.5)	119 (19.2)	37 (6.0)
PC19	36 (5.8)	130 (21.0)	243 (39.2)	156 (25.2)	55 (8.9)
PC20	33 (5.3)	135 (21.8)	247 (39.8)	145 (23.4)	60 (9.7)
PC21	37 (6.0)	140 (22.6)	221 (35.6)	166(26.8)	56 (9.0)
PC22	22 (3.5)	87 (14.0)	245 (39.5)	213 (34.4)	53 (8.5)
PC23	10 (1.6)	74 (11.9)	236 (38.1)	216 (34.8)	84 (13.5)
PC24	14 (2.3)	82 (13.2)	212 (34.2)	232 (37.4)	80 (12.9)
PC25	17 (2.7)	87 (14.0)	220 (35.5)	204 (32.9)	92 (14.8)
PC26	26 (4.2)	116 (18.7)	263 (42.4)	170 (27.4)	44 (7.1)
PC27	17 (2.7)	112 (18.1)	231 (37.3)	206 (33.2)	54 (8.7)
PC28	127 (20.5)	120 (19.4)	199 (32.1)	142 (22.9)	32 (5.2)
PC29	189 (30.5)	180 (27.4)	156 (25.2)	88 (14.2)	17 (2.7)
PC30	87 (14.0.)	107 (17.3)	195 (31.5)	167 (26.9)	64 (10.3)

**Table 2:** Standardised items factor loading in the initial measurement model of POC-M

Second order factors	First-order factors	Items	Standardised item loading
Experiential/Cognitive processes	Consciousness-raising	PC1	0.821
		PC2	0.870
		PC3	0.711
	Dramatic relief	PC4	0.634
		PC5	0.648
		PC6	0.468
	Self-reevaluation	PC7	0.640
		PC8	0.723
		PC9	0.801
	Environmental reevaluation	PC10	0.775
		PC11	0.776
		PC12	0.768
	Self-liberation	PC13	0.729
		PC14	0.744
		PC15	0.554
Behavioural processes	Counter-conditioning	PC16	0.691
		PC17	0.830
		PC18	0.786
	Helping relationships	PC19	0.771
		PC20	0.847
		PC21	0.855
	Reinforcement management	PC22	0.723
		PC23	0.770
		PC24	0.761
	Social Liberation	PC25	0.762
		PC26	0.710
		PC27	0.738
	Stimulus Control	PC28	0.842
		PC29	0.660
		PC30	0.739

**Table 3:** Goodness of fit indices for the measurement models of POC-M (initial and final models)

Model	CFI	TLI	SRMR	RMSEA (90% CI)	RMSEA (*P*-value)
Initial model	0.880	0.867	0.075	0.058 (0.054, 0.062)	< 0.001
Final model^a^	0.922	0.911	0.064	0.048 (0.044, 0.051)	0.847

Notes: CFI = Comparative fit index; TLI = Tucker-Lewis index; SRMR = standardised root mean square residual; RMSEA = root mean square error of approximation; CI = confidence interval

a Model with correlated items’ residual and first order factors residual: PC7 with PC5, PC3 with PC1, PC5 with PC4, PC29 with PC17, PC18 with PC16, PC12 with PC2, PC8 with PC7; stimulus control with counter-conditioning, self-reevaluation with consciousness raising, stimulus control with social liberation and stimulus control with help relationships

**Table 4:** Standardised items’ loading, composite reliability and average variance extracted for the final measurement model of POC-M

Second order factors	Items	Standardised item loading	CR	AVE
Experiential/Cognitive processes	PC1	0.911	0.85	
	PC2	0.787		
	PC3	0.809		
	PC4	0.851		
	PC5	0.842		
	PC6	0.403		
	PC7	0.579		
	PC8	0.677		0.54
	PC9	0.840		
	PC10	0.775		
	PC11	0.782		
	PC12	0.762		
	PC13	0.736		
	PC14	0.740		
	PC15	0.549		
Behavioural processes	PC16	0.760	0.86	
	PC17	0.778		
	PC18	0.850		
	PC19	0.770		
	PC20	0.846		0.57
	PC21	0.856		
	PC22	0.721		
	PC23	0.767		
	PC24	0.766		
	PC25	0.755		
	PC26	0.710		
	PC27	0.747		
	PC28	0.824		
	PC29	0.677		
	PC30	0.724		
